# Abrogation of neutrophil inflammatory pathways and potential reduction of neutrophil-related factors in COVID-19 by intravenous immunoglobulin

**DOI:** 10.3389/fimmu.2022.993720

**Published:** 2022-10-20

**Authors:** Jorge Adrian Masso-Silva, George Sakoulas, Jarod Olay, Victoria Groysberg, Matthew Geriak, Victor Nizet, Laura E. Crotty Alexander, Angela Meier

**Affiliations:** ^1^ Section of Pulmonary and Critical Care, Veterans Affairs (VA) San Diego, La Jolla, CA, United States; ^2^ Division of Pulmonary, Critical Care, Sleep and Physiology, University of California San Diego (UCSD), La Jolla, CA, United States; ^3^ Department of Infectious Disease, Sharp Rees-Stealy Medical Group, San Diego, CA, United States; ^4^ Department of Pediatrics, School of Medicine, University of California San Diego, San Diego, CA, United States; ^5^ Division of Host-Microbe Systems and Therapeutics, Department of Pediatrics, University of California (UC) San Diego, La Jolla, CA, United States; ^6^ Department of Research, Sharp Healthcare, San Diego, CA, United States; ^7^ Skaggs School of Pharmacy and Pharmaceutical Sciences, University of California (UC) San Diego, La Jolla, CA, United States; ^8^ Department of Anesthesiology, Division of Critical Care, UCSD, La Jolla, CA, United States

**Keywords:** intravenous immunoglobulin (IVIG), neutrophils, NETosis, oxidative burst, COVID-19, corticosteroids, dexamethasone, tocilizumab

## Abstract

Pathogenesis of lung injury in COVID-19 is not completely understood, leaving gaps in understanding how current treatments modulate the course of COVID-19. Neutrophil numbers and activation state in circulation have been found to correlate with COVID-19 severity, and neutrophil extracellular traps (NETs) have been found in the lung parenchyma of patients with acute respiratory distress syndrome (ARDS) in COVID-19. Targeting the pro-inflammatory functions of neutrophils may diminish lung injury in COVID-19 and ARDS. Neutrophils were isolated from peripheral blood of healthy donors, treated *ex vivo* with dexamethasone, tocilizumab and intravenous immunoglobulin (IVIG) and NET formation, oxidative burst, and phagocytosis were assessed. Plasma from critically ill COVID-19 patients before and after clinical treatment with IVIG and from healthy donors was assessed for neutrophil activation-related proteins. While dexamethasone and tocilizumab did not affect PMA- and nigericin-induced NET production *ex vivo*, IVIG induced a dose-dependent abrogation of NET production in both activation models. IVIG also reduced PMA-elicited reactive oxygen species production, but did not alter phagocytosis. COVID-19 patients were found to have elevated levels of cell-free DNA, neutrophil elastase and IL-8 as compared to healthy controls. Levels of both cell-free DNA and neutrophil elastase were lower 5 days after 4 days of daily treatment with IVIG. The lack of impact of dexamethasone or tocilizumab on these neutrophil functions suggests that these therapeutic agents may not act through suppression of neutrophil functions, indicating that the door might still be open for the addition of a neutrophil modulator to the COVID-19 therapeutic repertoire.

## Introduction

Neutrophils are the most abundant immune cells in circulation and are key for host immune responses. Neutrophils are promptly recruited to sites of infection and key in shaping adaptive immune responses (1, 2). Despite their vital role in clearing infections, neutrophils can cause significant collateral tissue damage if not properly controlled (3, 4). This is evidenced by the pathologic role of neutrophils in many infections and autoimmune diseases (5–7). In the context of infections, our research and others recently identified neutrophilia and neutrophil activity as prognostic factors in COVID-19 and associated with increased disease severity (8). Acute respiratory distress syndrome (ARDS) is the heterogeneous condition at the center of COVID-19 pathophysiology, where inflammatory cells such as neutrophils are recruited to the lung, contributing to tissue damage, ultimately leading to respiratory failure (8–11).

As phagocytes, neutrophils engulf pathogens within phagosomes, merge them with lysosomes, leading to killing of pathogens *via* oxidative burst (producing reactive oxygen species or ROS), low pH, antimicrobial molecules and degrading enzymes (12). Another key antimicrobial function is the production of neutrophil extracellular traps (NETs), which are formed by expulsion of DNA with adherent antimicrobial proteins including myeloperoxidase (MPO), elastase, histones, LL-37, etc. (13). In addition, neutrophils can release ROS, which can modify extracellular targets and affect the function of neighboring cells (14). Both NETosis and oxidative burst have been implicated in tissue damage (3–5) and are highly pro-inflammatory (4, 13, 14).

Corticosteroids are commonly used to treat inflammatory diseases to limit tissue damage (*e.g.* bacterial meningitis, brain tumors, and connective tissue diseases) (15). Dexamethasone is a widely used corticosteroid and became a standard treatment during the COVID-19 pandemic. Dexamethasone was found to lower 28-day mortality among COVID-19 patients who were receiving either invasive mechanical ventilation or oxygen alone (16, 17). Other therapeutic approaches in COVID-19 involve the use of biologics that target pro-inflammatory cytokines, such as IL-6, IL-1, GM-CSF, and TNFα (18). Tocilizumab, a monoclonal antibody against the IL-6 receptor, was the first biologic agent with proven efficacy in COVID-19 and is now an approved therapy (18). However, a recent randomized controlled trial (RCT) found that tocilizumab does not improve clinical outcomes or decrease mortality at 28 days (19), although the findings of another RCT supports the use of tocilizumab for patients with moderate-to-severe COVID-19 and high c-reactive protein (CRP) levels (20). Nevertheless, despite the implementation of these and other therapies, COVID-19 mortality rate remains high in patients with critical illness (21).

Intravenous immunoglobulins (IVIG) have been used primarily in the treatment of autoimmune diseases, with favorable results. In many of these diseases, including systemic lupus erythematosus, antiphospholipid syndrome and multiple sclerosis, neutrophils have been found to be important drivers of exacerbations (22–25). Multiple small clinical trials of IVIG as a treatment in COVID-19 have shown beneficial effects by reducing mortality and time of hospitalization (26–34), while one RCT found that treatment with IVIG (added to hydroxychloroquine and lopinavir/ritonavir therapies for all trial subjects) found that earlier IVIG administration correlated with shorter hospital and ICU stays (35). On the other hand, the results of another large and randomized clinical trial have suggested that IVIG treatment may lead to increased mortality and increased frequency of serious adverse events (36).

IVIG modulates the activation of multiple leukocytes, including monocytes/macrophages (37, 38), dendritic cells (38), T cells (39) and B cells (40). Interestingly, *ex vivo* treatment of leukocytes with IVIG has been shown to reduce production of ROS (41). In the context of neutrophils, early studies found that IVIG can activate neutrophils, leading to production of ROS (42–44), release of MPO (45) and elastase (46), and alterations in surface markers (47). More recently, Bohländer *et al*, found that IVIG treatment decreases inflammation in neutrophil-like HL-60 cells *via* reduction in release of cytokines (48), and Uozumi et al. observed that IVIG treatment of human neutrophils led to reduced NETosis by fluorescence microscopy (49). However, there are significant differences in the experimental designs of all those studies.

Based on the proven anti-inflammatory effects of IVIG observed in patients with autoimmune diseases, the discrepancies across prior *ex vivo* and *in vitro* assessments of IVIG on neutrophil function, and our previous observation of the contribution of neutrophils to ARDS immunopathology in COVID-19 (8), we undertook this study to define the impact of IVIG and anti-inflammatory treatments used in COVID-19 (dexamethasone and tocilizumab) on key pro-inflammatory and antimicrobial neutrophil functions. Further, we assessed NET components within plasma of COVID-19 patients whose therapy was complemented with IVIG.

## Materials and methods

### Study design and oversight

Patients admitted to a single center in San Diego, CA with COVID-19 confirmed by a PCR test (nasal or pharyngeal sample) who developed rapid hypoxemic respiratory failure from ARDS and requiring mechanical ventilation were considered for off-label IVIG therapy (0.5g/kg adjusted body weight per day for 4 consecutive days) < 72 hours from onset of mechanical ventilation. Patients with any baseline chronic organ failure comorbidities (*e.g*. heart failure, renal failure, stroke, dementia, or active malignancy) were excluded. A protocol entitled “Pilot study of the use of IVIG in patients with severe COVID-19 requiring mechanical ventilation and to assess their biological responses to IVIG therapy” (clincaltrials.gov NCT04616001) allowed for patient or next of kin consent for analysis of blood samples for this study that were obtained before, during, and after IVIG therapy. Blood analyzed consisted of residuals from samples drawn as part of routine daily blood work. Other than receipt of off-label IVIG, patients received standard of care diagnostic and therapeutic management contemporary for the time in the pandemic (December 2020-March 2021). The study was reviewed and approved by the Sharp Healthcare Internal Review Board (#2010902). *Ex vivo* neutrophil and plasma studies were conducted with VASDHS institutional review board (IRB) approval B200003, a non-human subject research waiver from the UCSD Institutional Review Board (IRB), and in accordance with the Helsinki Declaration of the World Medical Association.

### Plasma and neutrophil collection

Blood from healthy donors or COVID-19 patients was drawn in heparinized tubes and plasma was isolated by centrifugation at 1000 x g for 10 minutes. Plasma was aliquoted and stored at -80°C. For neutrophil isolation, blood from healthy donors was drawn and layered onto Polymorphprep™ (PROGEN) per manufacturer’s instructions. Briefly, 20 ml whole blood was gently layered onto 20 ml of Polymorphprep™ in a 50 ml conical tube and centrifuged at 500 x g for 30 min at room temperature (RT), sans brake. The granulocyte layer was collected and washed with HBSS^Ca-/Mg-^ and centrifuged at 400 x g for 10 min at RT. Cell pellet was re-suspended in 1 ml of HBSS^Ca-/Mg-^ and cells were counted using an hemocytometer. Average neutrophil purity across samples was 90%. Finally, neutrophils were resuspended at 2x10^6^ cells/ml for functional assays.

### Drug preparations (IVIG, dexamethasone, and tocilizumab)

Up to 4 final concentrations of IVIG (Octagam^®^ 10% from Octapharma USA, Inc), were used (0.2, 1, 5 and 10 mg/ml). For dexamethasone, we used a 4 mg/ml stock (Fresenius Kabi) and diluted to 100, 500 and 1000 ng/ml. Tocilizumab was prepared from 20mg/ml (Actemra) and diluted to 20, 200 and 400 μg/ml. All diluted stocks were prepared in HBSS^Ca+/Mg+^.

### Quantification of Cell-Free DNA from plasma of patients and for ex vivo NETosis assays

Neutrophils (2x10^5^ in 100 µl HBSS^Ca+/Mg+^) were plated in 96-well plates and incubated at 37°C with 5% CO_2_ with and without IVIG for 30 min, followed by stimulation with either phorbol 12-myristate 13-acetate (PMA) at 25 nM or Nigericin at 15 μM. All conditions were run in triplicate. After 2 hours, 500 mU/ml of micrococcal nuclease was added to each well and incubated for 10 min at 37°C with 5% CO_2_. Next, after addition of EDTA, plates were centrifuged at 200 x g for 8 min, and 100 µl supernatant was collected and transferred to a fresh 96-well plate. For quantification of NETs (from *ex vivo* assay) and cell-free DNA (from plasma of patients) the Quant-iT™ PicoGreen™ dsDNA Assay Kit (Invitrogen) was used, following the manufacturer’s protocol. Fluorescence was measured using the Infinite M200 (TECAN) plate reader with 480ex/520em.

### Imaging of NETs

Neutrophils at 2x10^5^ in 100 µl/well were seeded in an 8-chamber glass slide (Nunc™ Lab-Tek™ II Chamber Slide™ System) and incubating at 37°C with 5% CO_2_ for 30 min with and without IVIG. Neutrophils were stimulated with 25 nM PMA and incubated at 37°C with 5% CO_2_ for 2 hours. All conditions were run in duplicate. Cells were fixed with 4% Paraformaldehyde and stored at 4°C overnight. To stain for NETs, wells were washed with 1x PBS and then blocked with 2% bovine serum albumin (BSA) and 2% goat serum in PBS for 45min. Cells were incubated for 1 hour with rabbit anti-human MPO primary antibody (1:300 in 2% PBS-BSA; Dako North America, Inc.), and then with Alexa Fluor 488 goat anti-rabbit IgG secondary antibody (1:500 in 2% PBS-BSA; Life Technologies) for 45 min. Finally, cells were washed and incubated 10 min with 1 μM Hoechst-33342-trihydrochloride. Three washes with 1x PBS were performed after each staining step. Images were obtained using a Zeiss AxioObserver D1 microscope equipped with an LD A-Plan 20x/0.45 Ph1 and 10x/0.30 Ph1 objectives.

### Quantification of oxidative burst

We placed 1.2 ml of 2x10^6^ neutrophils/ml in 1.5 ml siliconized tubes and incubated with 10 μM 2’,7’-dichlorodihydrofluorescein diacetate for 20 min at 37°C on an orbital shaker. Cells were centrifuged at 500 x g for 8 min and resuspended in the original volume with HBSS^Ca-/Mg-^. Neutrophils at 2x10^5^ in 100 µl were seeded in a 96-well plate and pre-treated with IVIG or dexamethasone for 30 min. Cells were then stimulated with 25 nM of PMA. Each condition was run in triplicate. Cells were incubated at 37°C and 5% CO_2_ and fluorescence measured using an Infinite M200 (TECAN) plate reader with 495ex/520em, every 15 min for 2 hours.

### Quantification of phagocytosis

Neutrophils were seeded in a 96-well plate at 2x10^5^ per well. Cells were pre-treated with IVIG or dexamethasone for 30 min and cells were washed once with 1x HBSS^Ca-/Mg-^ to remove IVIG or dexamethasone prior to adding bioparticles. Cells were resuspended at 1:1 with pHrodo™ Red *Staphylococcus aureus* Bioparticles™ (Invitrogen) diluted in HBSS^Ca+/Mg+^, centrifuged at 1200 rpm for 5 min and incubated at 37°C and 5% CO_2_. Fluorescence was measured with 560ex/585em every 15 min for 2 hours. To exclude background signal, fluorescence of wells containing either HBSS, cells and pHrodo™ Red *S. aureus* Bioparticles was also measured.

### Quantification of NET and neutrophil activation related markers in the plasma of COVID-19 subjects

To measure granulocyte-derived elastase, plasma at 1:100 was assessed *via* the PMN Elastase Human ELISA kit (Abcam), following the manufacturer’s protocol. For quantification of MPO-DNA, a 96 well plate was coated with 50μl/well of.01 μg/ml anti-MPO mAb (Upstate) and incubated overnight at 4°C. Wells were washed twice with 1x PBS and blocked with 2% BSA for 2 hours. Wells were washed three times and 100μl of plasma samples were added at 1:4 dilutions and incubated at 4°C overnight. Peroxidase-labeled anti-DNA mAb (Cell Death ELISAPLUS, Roche) at a dilution of 1:25 in 100μl of assay buffer was added and plates incubated for 2 hours with shaking at 300 rpm at RT. Wells were washed 3 times and 100 μl of peroxidase substrate was added and incubated for 20 min at RT in the dark. The reaction was stopped with 50μl of 160mM sulfuric acid, and absorbance measured at 405nm. IL-8 was quantified from plasma at a 1:2 dilution using the DuoSet human IL-8/CXCL8 ELISA kit (R&D Systems) following the manufacturer’s instructions.

### Statistical analysis

Statistical analyses were performed using GraphPad Prism Version 8.4.3 (San Diego). Plasma cytokine levels and NETosis in controls and COVID-19 subjects were analyzed with Mann-Whitney tests. ROS production and phagocytosis assays were analyzed using a 2-way ANOVA with Geisser-Greenhouse correction and Dunnett’s multiple comparisons test for each timepoint. Two-sided tests and α level of 0.05 were used to determine significance.

## Results

### IVIG diminishes NETosis

**Figure 1:**
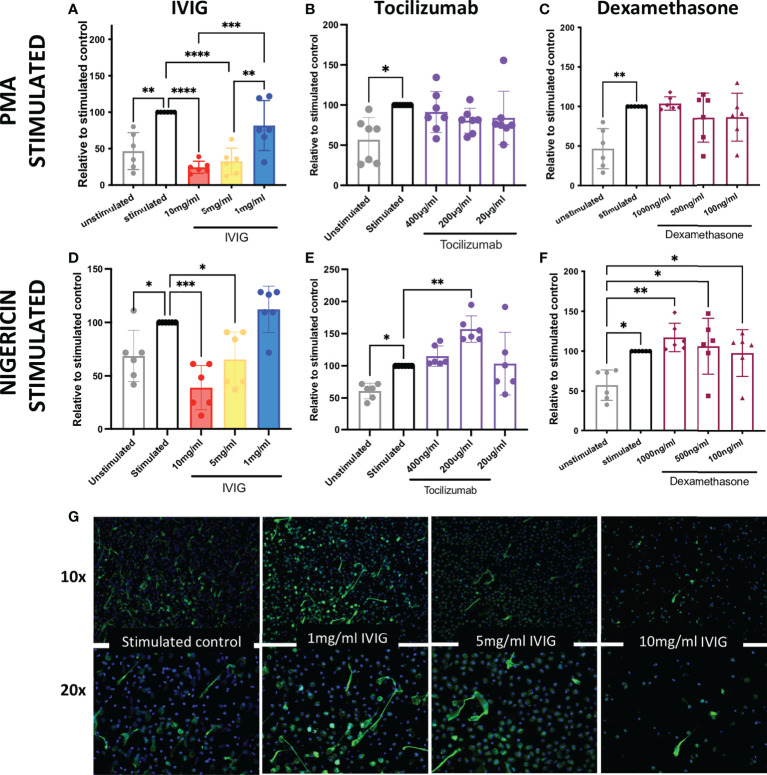
IVIG has a dose-dependent suppressive effect on NETosis, which suggests that it may be a complimentary treatment in acute lung injury. *Ex vivo* functional assays of healthy neutrophils stimulated with either PMA or nigericin shows that abrogation of NETosis is induced in a dose-dependent manner by: IVIG **(A, D)**, but not tocilizumab **(B, E)** or dexamethasone **(C, F)**. An alternative approach of assessing NETosis using fluorescence microscopy of neutrophils stained for myeloperoxidase (green; a key component of NETs) shows clear inhibition of NETosis by IVIG **(G)**. Data in panels A–F was analyzed with one-way ANOVA with multiple comparisons. Data are presented as individual data points ± SD with n = 6–7 individuals per group. Error bars represent 95% confidence interval. *P < .05, **P < .01, ***P < .001, ****P < .0001.

In order to assess the potential mechanistic effects of IVIG on neutrophil functions, and thus identify further immunomodulatory properties of IVIG, we pre-treated primary human neutrophils with IVIG prior to inducing NETosis. Our assays were performed with isolated neutrophils from healthy donors with over 90% of both live cells (defined at the end of assay in untreated cells) and purity (**Supplementary Figures 1A, B**). Pre-treatment with IVIG leads to a significant reduction in NETosis, which was dose-dependent after activation of NETosis through different pathways since both PMA- and nigericin-activated neutrophils abrogated NETosis when treated with IVIG (**Figures 1A, D**). In addition, treatment with IVIG was also able to abrogate spontaneous NETosis on unstimulated neutrophils (**Supplementary Figure 2A**). On the other hand, therapeutic concentrations of tocilizumab (19) (**Figures 1B, E**) and dexamethasone (16) (**Figures 1C, F**) did not affect NETosis using both types of activation. Furthermore, concomitant exposure of neutrophils to IVIG and dexamethasone does not show a synergistic effect on reducing NETs (**Supplementary Figure 2B**). Upon visualizing NETs *via* fluorescence microscopy, similar findings were seen, with reduction of NETs by IVIG treatment visualized by staining of MPO (a major component of NETs) in a dose-dependent manner (**Figure 1G**).

### IVIG diminishes neutrophil oxidative burst

**Figure 2:**
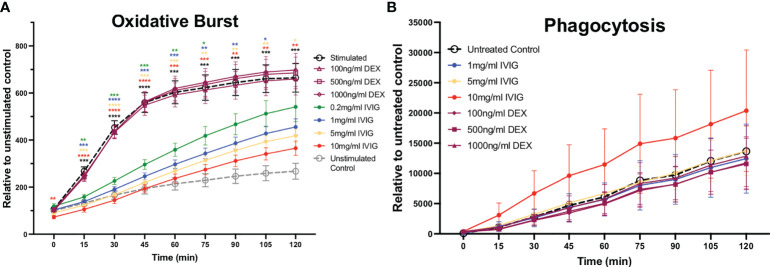
Treatment of primary human neutrophils with IVIG inhibits oxidative burst in a dose-dependent manner, while preserving phagocytosis. Circulating neutrophils from healthy controls were used to assess antimicrobial functions including reactive oxygen species production (oxidative burst) and phagocytosis. **(A)** Neutrophil oxidative burst, while being an important antimicrobial function can cause significant collateral damage to tissues, was inhibited in a dose-dependent manner by IVIG (solid circle markers) but not by dexamethasone (hollow purple markers). **(B)** Neutrophil phagocytosis was retained (unaffected) by treatment with IVIG or dexamethasone. Data were analyzed with a 2-way ANOVA with Geisser-Greenhouse correction and Dunnett’s multiple comparisons test. Data are presented as individual data points ± SEM with n = 9 and n = 5 individuals per group in oxidative burst and phagocytosis assays, respectively. Error bars represent 95% confidence interval. *P < .05, **P < .01, ***P < .001, ****P < .0001.

The production of ROS is a key mechanism used to kill pathogens and is involved in NETosis, which leads to significant inflammation in host tissues. Similar to the impact on NETosis, pre-treatment of neutrophils with IVIG led to reduced production of ROS in a dose-dependent manner, with the highest concentration tested (10mg/ml of IVIG) approaching the low levels of ROS observed in unstimulated controls (**Figure 2A**). Pre-treatment of neutrophils with varying concentrations of dexamethasone did not impact ROS production (**Figure 2A**).

### Treatment with IVIG preserves neutrophil phagocytosis

Besides NETosis and ROS production, neutrophils also utilize phagocytosis as a primarily antimicrobial mechanism. In the context of COVID-19, we previously found that neutrophils circulating in the blood of critically ill COVID-19 patients had increased phagocytic activity relative to healthy controls (8). Preserving or even increasing phagocytosis is important in COVID-19 and across ARDS patients in general, as these patients are at risk for opportunistic and nosocomial infections. In these studies, we found that neither treatment with IVIG nor dexamethasone had a significant effect on the phagocytic activity of neutrophils (**Figure 2B**). It is important to mention that IVIG was removed from the assay prior adding the *S. aureus* bioparticles in order to focus on intrinsic changes to neutrophil phagocytic activity, since IVIG can opsonize *S. aureus* bioparticles thus leading to increased phagocytosis.

### IVIG treatment and neutrophil-related factors in the circulation of COVID-19 patients

**Figure 3:**
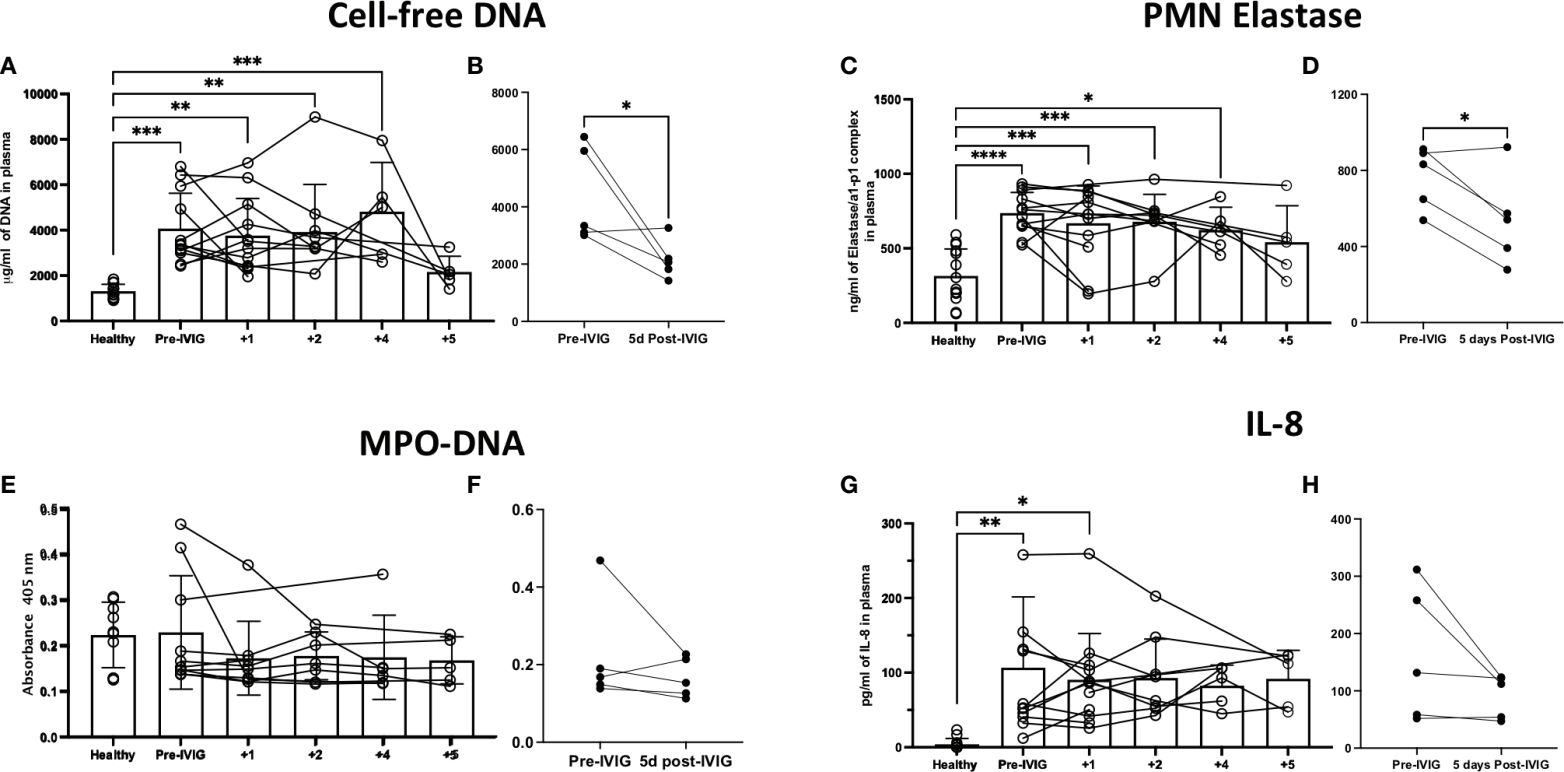
Treatment of severe COVID-19 with IVIG may lead to decreased NET-related factors and IL-8 in the circulation of patients. We assessed NET related components in the circulation of IVIG-treated COVID-19 patients, hours before the first IVIG dose and up to 5 days after treatment with IVIG. Moreover, we did a paired comparison of pre-IVIG plasma versus 5 days post-IVIG. We assessed NET components such as cell-free DNA **(A, B)**, neutrophil elastase **(C, D)**, and myeloperoxidase (MPO)-DNA complexes **(E, F)**. Cell-free DNA and neutrophil elastase were significantly elevated in COVID-19 patients relative to controls **(A, C)**, and decreased after IVIG treatment **(B, D)**. MPO-DNA was not elevated in the plasma of patients with severe COVID-19 and did not change after IVIG treatment **(E, F)**. The neutrophil chemokine and activating cytokine IL-8 was elevated in the plasma of COVID-19 patients **(G)** but did not significantly decrease after IVIG treatment **(H)**. Data for panels A, C, E, and G was analyzed *via* one-way ANOVA with Tukey’s multiple comparisons test, although data points from same patients are connected to observe data trends. Data for panels B, D, F and H was analyzed with paired t-tests. Data are presented as individual data points ± SD with healthy controls (n = 10-14) and COVID-19 (n = 12). Error bars represent 95% confidence interval. *P < .05, **P < .01, ***P < .001, ****P < .0001.

Cell-free DNA and PMN elastase were significantly elevated in COVID-19 patients prior to IVIG treatment, as compared to healthy controls (**Figures 3A, C**, respectively). After 5 days of IVIG treatment, both cell-free DNA and PMN elastase were significantly reduced (**Figures 3B, D**, respectively). MPO-DNA complexes were not increased in COVID-19 subjects relative to healthy controls, and did not change across time or when compared prior to the first IVIG dose versus 5 days later (**Figures 3E, F**, respectively). Finally, IL-8, a key cytokine for neutrophil activation and recruitment, was elevated in COVID-19 patients as compared to healthy controls (**Figure 3G**), with the IL-8 level at 5 days post IVIG treatment having a non-significant downward trend (**Figure 3H**).

## Discussion

Neutrophils have a “dark side” capable to induce significant immunopathology in different inflammatory settings (3, 4, 7). In the context of COVID-19, a wide variety of studies established neutrophils and their activity with detrimental effects during the disease (8, 10). In COVID-19 and other respiratory diseases, avoiding the development of ARDS is critical (50). ARDS is a very heterogeneous clinical picture with no specific treatment and management of ARDS patients is reduced to supportive therapy (9, 51). During ARDS development, inflammatory cells cause tissue damage affecting lung function (9). Neutrophils have been associated with the development of ARDS in COVID-19 and to worsen the prognosis of the disease (8, 10, 11).

During the course of COVID-19, it is crucial to avoid exacerbation of inflammation, which can lead to organ failure, including ARDS. Thus, the use of corticosteroids in COVID-19 has been widely used worldwide (52). Dexamethasone is one of the most commonly used corticosteroid to diminish and control inflammation in the context of COVID-19 (16, 52). Clinical data supports the use of dexamethasone in patients receiving either invasive mechanical ventilation or oxygen alone (reducing 28-day mortality), although not in those receiving no respiratory support (16). This suggests that clinical situation of the patient and the timing of intervention during the disease course is important to achieve positive results. In the context of neutrophils and their potential contribution to exacerbation of inflammation, here, we show through our established *ex vivo* functional assays that dexamethasone does not have effect in three major neutrophil pathways associated to inflammation, NETosis, oxidative burst and phagocytosis (**Figures 1C**, **2A, B**, respectively). It has been shown that dexamethasone can inhibit *S. aureus*-induced NETosis but not PMA-induced, although that study used a concentration 4 times higher (10μM, thus around 4000 ng/ml) than our highest concentration (1000ng/ml) (53), reaching levels significantly higher that are not probably achieved physiologically during COVID-19 treatment, which is usually 6 mg of dexamethasone per day (23). Recently, it has been reported that in the context of COVID-19, dexamethasone can downregulate interferon-stimulated genes and activated IL-1R2^+^ in neutrophils, and to expand immunosuppressive immature neutrophils (54), thus the mechanism by which dexamethasone can diminish neutrophil activity is by affecting their functions toward a immunosuppressive phenotype. Similar to dexamethasone, tocilizumab, a monoclonal antibody against the interleukin-6 receptor (IL-6R) used to abrogate IL-6 signaling, does not have any effect in NETosis when used within the range of therapeutic concentrations (**Figure 1C**). Although, tocilizumab are also immunoglobulins as IVIG, tocilizumab in a monoclonal population of IgGs that are used at a lower concentrations than IVIG due to its specificity for the IL-6R, hence the effect on neutrophils may not be achieve at the used concentrations and by the nature of formulation of tocilizumab. On the other hand, IVIG is able to significantly abrogate NETosis (under both PMA and nigericin activation) and oxidative burst in a dose-dependent manner (**Figures 1A, D, G**, **2A**), which suggests that complementation of standard treatment with IVIG might account for the lower mortality rate observed in IVIG-treated patients through diminishing neutrophil-mediated immunopathology, having implications in other conditions with neutrophil-mediated immunopathology. Interestingly, neither dexamethasone, tocilizumab nor IVIG seem to have an effect in changing the phagocytic activity of neutrophils. This data concur with our previous findings showing that neutrophils isolated from COVID-19 have enhanced phagocytic activity as compared to healthy controls, even when some COVID-19 patients received dexamethasone and/or tocilizumab (8). IVIG have shown to be able to modulate neutrophil functions, although in contrary to our results, early studies showed that IVIG can induce the production of ROS (42–44), NETs (myeloperoxidase and elastase) (45, 46) and increased activation status defined by changes in surface markers (47). However, recently, Bohländer et al, found that IVIG can decrease inflammation in the neutrophil-like HL60 cells by reducing the release of cytokines such as IL-10, MCP-1 and IL-8 (48); and Uozumi et al. observed that treatment of human neutrophils with 5ml/ml of human sulfo-immunoglobulins lead to decreased production of NETs defined by staining of DNA with SYTOX (49), a different approach that this study for NET assessment. Nevertheless, there are significant differences in the experimental designs of all those studies. For instance, although most studies used isolated neutrophils (31, 43–45), some studies used whole blood (47) or a neutrophil-like cell line (48). Moreover, neutrophil activation varied from using live bacteria (15, 45), LPS (47), TNFα (21, 46) and even SARS-CoV-2-like particles associated to SARS-CoV-2 spike protein specific IgG, IgA, and IgM antibodies (48). In this study, we used PMA, which is commonly used in leukocyte activation studies, and studying neutrophils is not the exception, particularly in assessing NETosis and oxidative burst (55, 56).

IVIG has been used for a while to treat autoimmune diseases, evidencing its immunomodulatory properties (25). Hence, early attempts to introduce IVIG to counteract hyperinflammation in COVID-19 were expected. However, contradictory conclusions have arisen, from being beneficial by reducing mortality and time of hospitalization (26–34), to showing no differences in mortality rate (35), to even suggesting a tendency to be associated with an increased frequency of serious adverse events (36). Nevertheless, differences between these studies may account for their opposite conclusions. For instance, there are differences in dosage of IVIG, length of treatment, timing of first dose upon admission, the formulation of IVIG, additional treatments, amount of subjects and subject heterogeneity.

Despite ambiguous results at the global level, our specific clinical experience with IVIG has been highly successful when niche-applied early to patients without end-organ comorbidities or advanced age (57, 58). As part of quality measurements by Sharp Healthcare, data was collected to assess clinical outcomes in patients with COVID-19 at different stages of the pandemic. Review of these data has demonstrated a survival benefit in IVIG recipients ≤70 years of age with severe to critical COVID-19 (receipt of high-flow oxygen or greater oxygen requirement, WHO severity score 5-7). In these patients, receipt standard of care alone (remdesivir, glucocorticoids) was 21%, receipt of adjunctive tocilizumab mortality was 23%, and only 7% in IVIG recipients (p=0.03, Fisher exact test, unpublished observations). Although limited in sample size and a single-center retrospective assessment, this clinical experience coupled with our findings showing IVIG attenuation of neutrophil-driven inflammation clearly sets the stage for important more rigorous future clinical study of IVIG. Interestingly, we previously showed that COVID-19 treated with tocilizumab have increased IL-6 in circulation (8), which correlates with a numerically higher mortality in tocilizumab recipients, and also aligns with a larger study showing no improvement on the clinical status or lower mortality (19).

The inconsistent results of IVIG benefit in COVID-19 rest largely on the broad heterogeneity of COVID-19 patient types. COVID-19 therapeutics are likely impactful only in younger patients at high-risk for COVID-19 disease with comorbidities like obesity, hypertension, and diabetes mellitus but without end-organ damage. In settings of advanced age and/or baseline organ failure, mortality from COVID-19 may be significantly less modifiable, not only due to direct COVID-19 factors, but due to the increased risk of secondary complications such as cardiovascular, thrombotic, or secondary bacterial or fungal infections, and even adverse drug reactions. Unfortunately, large prospective clinical trials may be underpowered to be able to address specific patient subtypes within their cohorts (36, 59). Thus, more studies are needed to consider IVIG as part of standard therapy for COVID-19 (and perhaps viral ARDS), specifically addressing subpopulations who may benefit. Related to this, there is evidence showing that the beneficial effects of IVIGs might depend on the composition of the IVIG preparation, the immune status of the patient, and the severity of the disease (60). Although the majority of the studies use IVIG based on IgGs, other studies have shown that IVIG containing relevant amounts of IgM and IgA (in addition to IgG) can modulate the release of pro-inflammatory cytokines in neutrophil-like HL60 cell line better than IVIG containing mostly IgGs (48). Nevertheless, in our cohort of patients we found that neutrophil-related factors, such as those involved in NETosis, including cell-free DNA and PMN elastase seem to be reduced upon complementation with IVIG (**Figures 3A–D**), although even when cell-free DNA is a well-accepted indicator of NET in circulation, it can also come from other sources such as dead cells. Moreover, MPO-DNA did not show significant differences although trend over time appears to be downward, and interestingly there was no difference between healthy donors and COVID-19 patients (**Figures 3E, F**). Similarly, IL-8, a strong chemoattractant and activator of neutrophils also showed a trend down over time, but in this case there was a significant difference between COVID-19 and healthy plasma (**Figures 3G, H**), which aligns to our previous findings and others associating increased IL-8 in the severity of COVID-19 (8).

Currently, different trials are attempting to inhibit or block neutrophil pathways to control inflammation. Several current trials are targeting NETosis, since they are highly pro-inflammatory (61). Some trials are addressing the use of DNase (61–64), which tackle NETs effects once they are released, (but may not directly inhibit proteolytic enzymes coating the DNA strands) in contrary to IVIG, which prevent their release according to our *ex vivo* assays. Related to this, a recent non-randomized open-label study evaluated the complementation with inhaled DNase plus tocilizumab and baricitinib (JAK1/2 inhibitor), finding association with lower in-hospital mortality and intubation rate, shorter duration of hospitalization, and prolonged overall survival; in addition, in an *in vitro* approach, plasma from those COVID-19 patients undergoing the complemented treatment induced less tissue factor/thrombin pathway in primary lung fibroblasts as compared to standard-of-care (64). Nevertheless, it is important to consider that the anti-inflammatory effect of IVIG also occurs in other leukocytes and that this study is strictly limited to neutrophils. Thus, more studies are needed to address the questions to the overall impact of IVIG in COVID-19 patients and other inflammatory conditions.

This study has limitations such as the small cohort of COVID-19 patients, the lack of some samples from each time point, limited to components in circulation (even when neutrophils are phenotypically different from circulation to lung tissue (65). Our *ex vivo* studies utilized two activation stimuli, which may not accurately reflect all NET compositions, as such compositions can change depending on the stimulus (66, 67). Furthermore, there was no comparator group of similar illness severity and similar points of illness who did not receive IVIG or received placebo in its place. Regardless of the limitations, this study established the foundation of the effect of IVIG in three key functions of neutrophils and along with data from IVIG-treated patients suggests that complementation of IVIG to standard therapy for COVID-19 may diminish neutrophil pro-inflammatory pathways, which are not affected by the commonly used corticosteroid, dexamethasone.

## Data availability statement

The raw data supporting the conclusions of this article will be made available by the authors, without undue reservation.

## Ethics statement

The study was reviewed and approved by the Sharp Healthcare Internal Review Board (#2010902). Ex vivo neutrophil and plasma studies were conducted with VASDHS institutional review board (IRB) approval B200003, a non-human subject research waiver from the UCSD Institutional Review Board (IRB), and in accordance with the Helsinki Declaration of the World Medical Association. The patients/participants provided their written informed consent to participate in this study.

## Author contributions

JM-S, AM, VN and LC contributed to conception and design of the study. JM-S and AM organized the database. JM-S, JO and VG performed the experiments. JM-S and LC performed the statistical analysis. JM-S wrote the first draft of the manuscript. GS wrote sections of the manuscript. GS and MG provide key samples. All authors contributed to manuscript revision, read, and approved the submitted version.

## Funding

This work was supported by a Veterans Affairs Merit Award 1I01BX004767 (LC), NIH NHLBI K24HL155884 and R01HL137052 (LC), TRDRP Award T30IP0965 (LC), and UCSD ACTRI 1KL2TR001444 (AM).

## Acknowledgments

Octagam 10% for this study both for patient administration and for *in vitro* work was provided by Octapharma USA (Hoboken, NJ).

## Conflict of interest

Author GS has received consulting and research fees from Octapharma USA (Hoboken, NJ).

The remaining authors declare that the research was conducted in the absence of any commercial or financial relationships that could be construed as a potential conflict of interest.

## Publisher’s note

All claims expressed in this article are solely those of the authors and do not necessarily represent those of their affiliated organizations, or those of the publisher, the editors and the reviewers. Any product that may be evaluated in this article, or claim that may be made by its manufacturer, is not guaranteed or endorsed by the publisher.

## Supplementary material

Supplementary Figure 1 Cell viability and purity of neutrophils for functional assays. Upon isolation of neutrophils, viability was assessed by staining with propidium iodide and analyze by flow cytometric analysis, observing over 90% live cells **(A)**. In addition, over 90% purity was obtained in isolated cells, which was observed by cytospining cells and staining with Giemsa, a representative picture is shown **(B)**.

Supplementary Figure 2 IVIG alone can inhibit spontaneous NETosis and dexamethasone does not show synergistic inhibitory effect with IVIG. Isolated neutrophils were incubated with increasing concentrations of IVIG for 3.5 h and no activation stimuli was added to cell culture, observing that IVIG can also diminish spontaneous NETosis in a dose-dependent manner. In addition, a potential synergistic effect was assessed by incubating neutrophils with IVIG and dexamethasone concomitantly; observing that dexamethasone at 500 ng/ml does not enhance NETosis abrogation caused by IVIG **(B)**. Data was analyzed with one-way ANOVA with multiple comparisons. Data are presented as individual data points ± SD with n=6 individuals per group. Error bars represent 95% confidence interval. **P<.01, ***P<.001, ****P<.0001.
