# Supplementary figures and images for: Abrogation of neutrophil inflammatory pathways and potential reduction of neutrophil-related factors in COVID-19 by intravenous immunoglobulin
Source: Front Immunol. 2022 Oct 20;13:993720. doi: 10.3389/fimmu.2022.993720 (PMC9632428; doi:10.3389/fimmu.2022.993720)

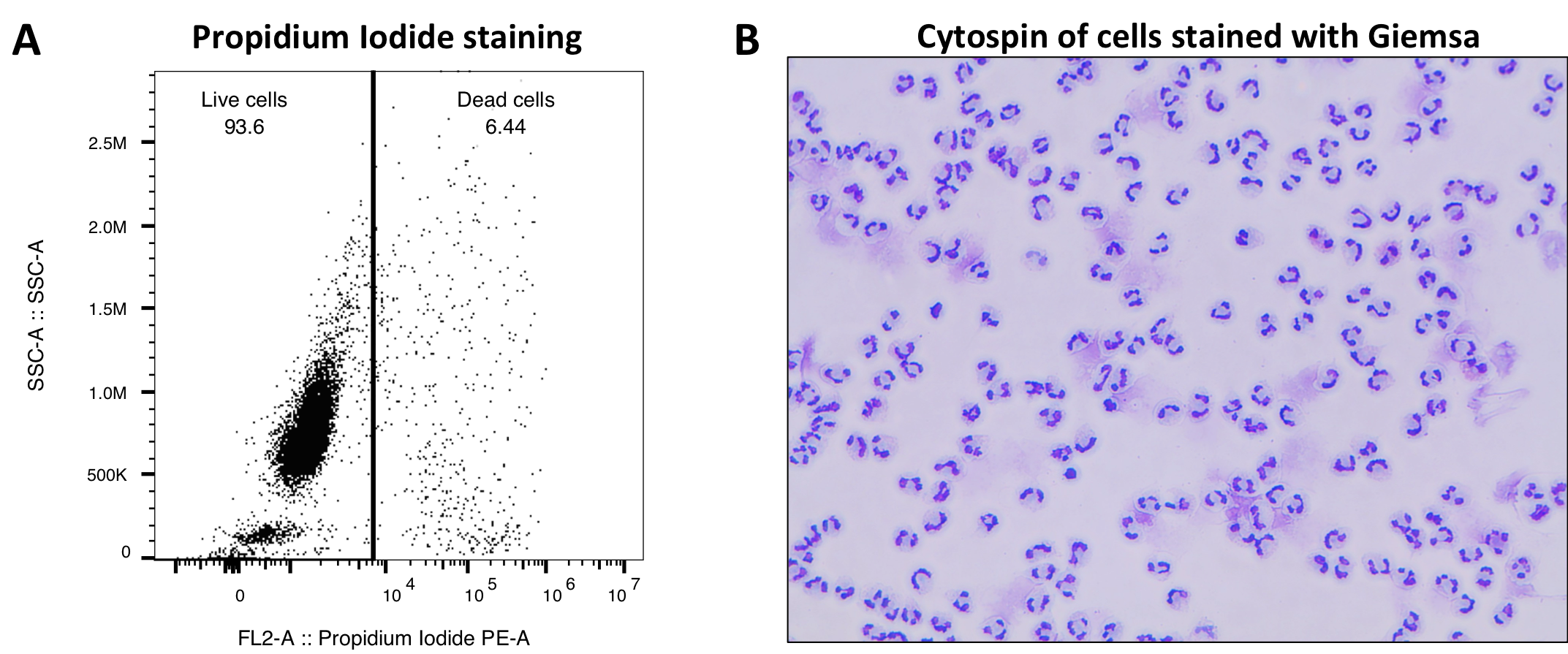

Supplement: Supplementary Figure 1 — Cell viability and purity of neutrophils for functional assays. Upon isolation of neutrophils, viability was assessed by staining with propidium iodide and analyze by flow cytometric analysis, observing over 90% live cells (A). In addition, over 90% purity was obtained in isolated cells, which was observed by cytospining cells and staining with Giemsa, a representative picture is shown (B). [file Image_1.tiff]

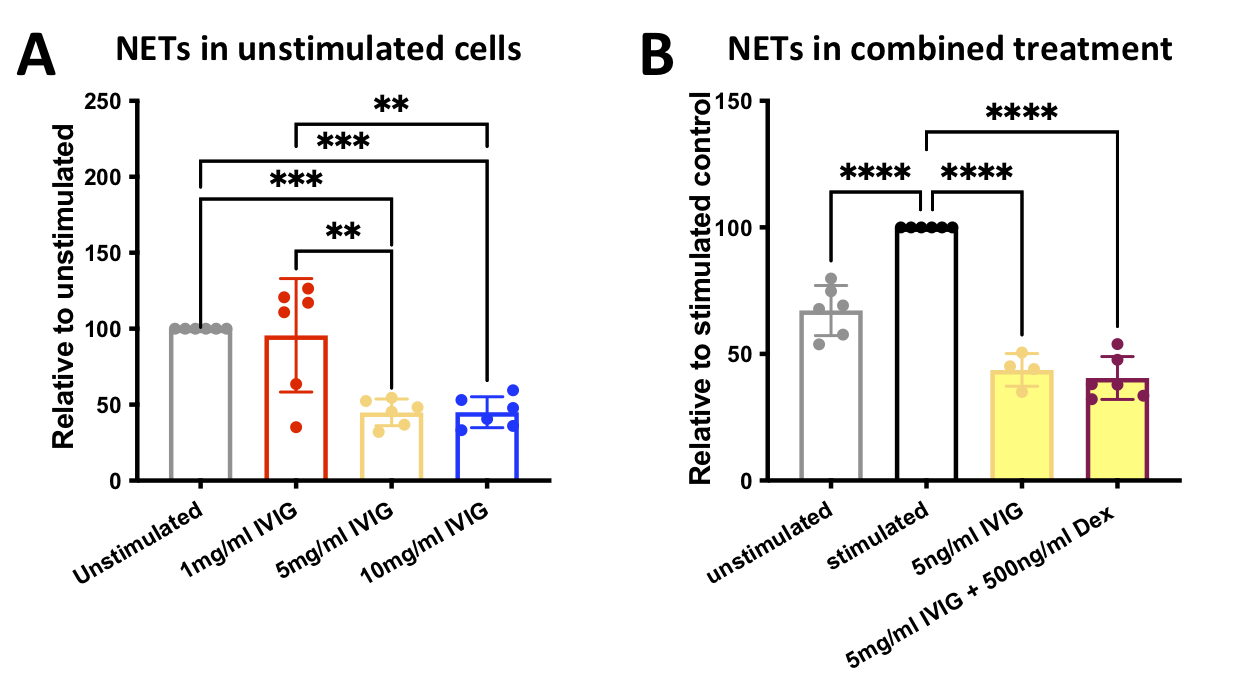

Supplement: Supplementary Figure 2 — IVIG alone can inhibit spontaneous NETosis and dexamethasone does not show synergistic inhibitory effect with IVIG. Isolated neutrophils were incubated with increasing concentrations of IVIG for 3.5 h and no activation stimuli was added to cell culture, observing that IVIG can also diminish spontaneous NETosis in a dose-dependent manner. In addition, a potential synergistic effect was assessed by incubating neutrophils with IVIG and dexamethasone concomitantly; observing that dexamethasone at 500 ng/ml does not enhance NETosis abrogation caused by IVIG (B). Data was analyzed with one-way ANOVA with multiple comparisons. Data are presented as individual data points ± SD with n=6 individuals per group. Error bars represent 95% confidence interval. **P<.01, ***P<.001, ****P<.0001. [file Image_2.tiff]
